# Methylenedioxymethamphetamine (MDMA, ‘Ecstasy’): Neurodegeneration *versus* Neuromodulation

**DOI:** 10.3390/ph4070992

**Published:** 2011-07-05

**Authors:** Elena Puerta, Norberto Aguirre

**Affiliations:** Department of Pharmacology, School of Pharmacy, University of Navarra, Irunlarrea 1, 31008 Pamplona, Spain; E-Mail: epuerta@alumni.unav.es (E.P.)

**Keywords:** 3,4-methylenedioxymethamphetamine (MDMA, ‘ecstasy’), 5-hydroxytryptamine (5-HT, serotonin), neurotoxicity

## Abstract

The amphetamine analogue 3,4-methylenedioxymethamphetamine (MDMA, ‘ecstasy’) is widely abused as a recreational drug due to its unique psychological effects. Of interest, MDMA causes long-lasting deficits in neurochemical and histological markers of the serotonergic neurons in the brain of different animal species. Such deficits include the decline in the activity of tryptophan hydroxylase in parallel with the loss of 5-HT and its main metabolite 5-hydoxyindoleacetic acid (5-HIAA) along with a lower binding of specific ligands to the 5-HT transporters (SERT). Of concern, reduced 5-HIAA levels in the CSF and SERT density have also been reported in human ecstasy users, what has been interpreted to reflect the loss of serotonergic fibers and terminals. The neurotoxic potential of MDMA has been questioned in recent years based on studies that failed to show the loss of the SERT protein by western blot or the lack of reactive astrogliosis after MDMA exposure. In addition, MDMA produces a long-lasting down-regulation of SERT gene expression; which, on the whole, has been used to invoke neuromodulatory mechanisms as an explanation to MDMA-induced 5-HT deficits. While decreased protein levels do not necessarily reflect neurodegeneration, the opposite is also true, that is, neuroregulatory mechanisms do not preclude the existence of 5-HT terminal degeneration.

## 1. Introduction

**Figure 1:**
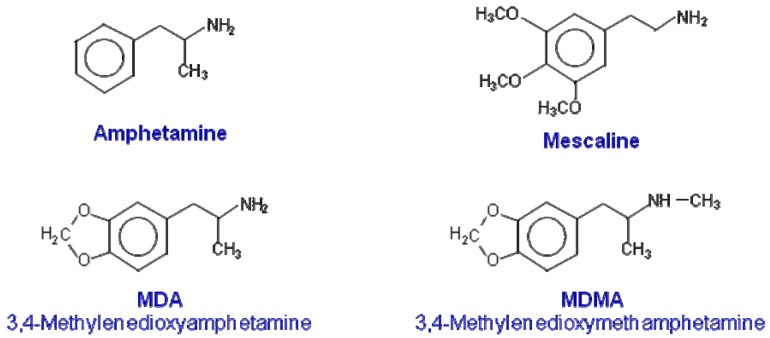
Chemical structures of MDMA and related compounds.

3,4-Methylenedioxymethamphetamine (MDMA or ecstasy) is a phenylpropanolamine with structural similarities to both amphetamine and mescaline (Figure 1). Ecstasy has come to symbolize one of the largest youth subcultures of the late 20th century and has established itself as one of the most popular recreational drugs of abuse [1,2]. The appeal of MDMA is related to its unique profile of psychotropic actions, which includes amphetamine-like stimulant effects, coupled with feelings of increased emotional sensitivity and closeness to others [3-9]. Based on this, Nichols [10] postulated that MDMA represents a novel class of compound, in that it possesses unique effects, which means that it cannot be classified as either a hallucinogen or a psychostimulant. Nichols [10] proposed the term “Entactogen” to describe the effects of MDMA and related compounds. This definition is based on the claimed ability of MDMA to allow therapists and patients to access and deal with repressed painful emotional issues [11]. Due to these unique psychological effects, although never marketed for this purpose, MDMA was used in the 70s and 80s by U.S. psychotherapists [4]. Nevertheless, concern over its increasing abuse and its possible neurotoxicity in humans led to the assignment of MDMA as a Schedule I agent by the U.S. Drug Enforcement Agency in 1985 [12]. Since then, there have been ongoing controversies regarding whether MDMA is medically useful as an adjunct in psychotherapy [13-17] and whether the neurotoxic findings seen after single or repeated high doses of MDMA in experimental animals are relevant for humans [18-23].

## 2. Pharmacology

As an in-depth examination of the preclinical pharmacology of MDMA is beyond the scope of this mini-review, only a brief summary will be detailed here (for more in-depth reviews, see [24-27]). Preclinical data gathered from research in laboratory animals, namely in rats, indicate that upon systemic administration, MDMA affects peripheral and central nervous system (CNS) functions by acting mainly on monoaminergic systems. Thus, initial studies showed that MDMA and its main metabolite 3,4-methylenedioxyamphetamine stimulate efflux of [^3^H]5-hydroxytryptamine (5-HT) [28] (Figure 1) and [^3^H]dopamine from preloaded synaptosomes [29,30]. Subsequent reports, using *in vivo* microdialysis, demonstrated that MDMA increases extracellular 5-HT, dopamine and noradrenaline levels in multiple brain regions with effects on 5-HT being greater in magnitude [31-34]. MDMA also enhances the release of acetylcholine, an effect that appears to be secondary to the activation of serotonergic, dopaminergic and/or histaminergic receptors [35]. The evaluation of monoamine release after MDMA intake has not been studied in humans or non-human primates, but several studies suggest that the interaction of MDMA with the 5-HT carrier and a subsequent release of 5-HT could be responsible for most of the physiological and psychological responses to MDMA in humans [5-8,36].

Available evidence also indicates that acute release of dopamine caused by MDMA mediates its reinforcing effects although non-monoaminergic effects may also be involved. It has also been shown that repeated MDMA induces behavioral sensitization and cross-sensitization to cocaine, MDMA substitutes for cocaine in a drug discrimination task, and that MDMA pre-exposure facilitates cocaine conditioned place preference and intravenous self-administration [37-41]. Despite these findings, a general consensus of the animal literature is that MDMA is a low-efficacy reinforcer when compared to self-administration of other drugs of abuse and does not possess the same addictive potential as stimulants like cocaine or methamphetamine [37]. In a similar fashion, the dependence potential of MDMA is much weaker than that reported for other drugs of abuse (e.g. opioids, alcohol). While some people report problems controlling and concern about their use, the notable lack of case reports of severe withdrawal syndromes in the literature suggests that physical symptoms play a more limited role than psychological ones [42]. Of interest, diminished subjective effects over repeated uses have often been reported by human ecstasy users [43], an effect that has been replicated in self-administration experiments with MDMA conducted in rhesus monkeys over an 18-month period of contingent drug exposure [39].

## 3. Toxicology

It is worth noting that MDMA-related medical complications have risen more than 20-fold in recent years in the U.S.A. and Europe, consistent with increasing popularity of the drug [44-46]. Serious adverse effects of MDMA intoxication include cardiac arrhythmias, hypertension, hyperthermia, serotonin (5-HT) syndrome, hyponatremia, liver complications, seizures, coma, and death [45]. However, considering the widespread use of MDMA, fatal intoxications remain rare events [2]. Further, accumulating evidence also indicates that long-term MDMA abuse is associated with cognitive impairments and mood disturbances, which can last for months after cessation of drug intake [47-54].

In animals, MDMA can cause long-lasting changes in neurochemical and histological markers of serotonergic function in brains of rats [18,19,55], primates [56-57] and, possibly, humans [58]. Such effect is evidenced by the decline in the activity of tryptophan hydroxylase [59]; a decrease in the content of 5-HT and its main metabolite 5-hydoxyindoleacetic acid (5-HIAA) [18,19,22,60,61]; a lower density of [^3^H]paroxetine-labelled 5-HT transporters (SERT) [20,62] along with the loss of SERT protein in several regions of the brain [63,64]; and long-term altered responses to 5-HT agonists or 5-HT releasing drugs in rats, non-human primates and MDMA users [65-69]. This constellation of findings, coupled with neuroanatomic observations using different techniques such as immunohistochemistry [63,70-72] and silver impregnation methods [73], strongly suggest that MDMA damages 5-HT terminals in rats and non-human primates [reviewed by 23]. It should noted, however, that in the study using the Fink-Heimer method [73], MDMA was administered to rats at a extremely high dose (80 mg/kg of twice a day for two days), a treatment that depleted not only striatal 5-HT but also dopamine content, making it difficult to know if degenerating terminals were dopaminergic or 5-HTergic. There have been no similar observations of long-term damage to other neurotransmitter systems in either rat or primate (except for [73]). Therefore, MDMA is considered to be a selective serotonergic neurotoxin. Of interest, all these effects are limited to 5-HT terminals of the serotonergic neurotransmitter system, as the cell bodies in the raphe nuclei are not damaged by MDMA [72,74].

Of interest, MDMA has a different pharmacology in the mouse compared to other laboratory animals. Thus, it has been repeatedly reported that MDMA is a relatively selective dopamine neurotoxin in mice, leaving 5-HT concentrations intact, in contrast to its selective 5-HT neurotoxicity in rats or non-human primates. Such neurotoxicity is evidenced by a decrease in the content of striatal dopamine and its main metabolites [75-77]; the decline in l-tyrosine hydroxylase and dopamine transporter immunostaining [78]; and increased markers of microglial and astrocytic activation in strict anatomical correlation with dopaminergic deficits [79]. It is noteworthy that these latter authors were the first to show that MDMA in mice causes a persistent loss of dopaminergic cell bodies in the substantia nigra, indicating that MDMA neurotoxicity in this animal species is not restricted to the loss of neuronal 5-HT terminals as it is in rats [79].

Undoubtedly, determining whether, under what circumstances, and to what extent MDMA exposure produces chronic changes in human brain function is a critical public health issue, specially because recently, clinical studies investigating the efficacy of MDMA-assisted psychotherapy in patients with a diagnosis of posttraumatic stress disorder have been approved in several countries worldwide and some other protocols are under review (the MAPS website should be consulted for further information http://www.maps.org/home/). Accordingly, a number of investigators have tested whether MDMA abuse results in neurotoxicity in the human brain [22, 60,80-88]. Some of these studies concluded that MDMA might be also toxic to humans since 5-HIAA levels in the CSF are reduced in MDMA abusers [22,60,80,83,84]. Recent advances in neuroimaging techniques have been applied to the study of the 5-HT system in the brains of humans with a history of MDMA abuse. Positron emission tomography or single photon emission computed tomography used in combination with a SERT ligand also found a lower density of brain 5-HT transporter sites in MDMA users [58,82,89-91].

Although the data summarized above would seem to present a solid case for neurotoxic effects of MDMA corresponding adverse functional consequences in human ecstasy users, such conclusions have been questioned for a number of reasons. These include inconsistent findings with respect to presence, magnitude and regional extent of brain areas affected and of persistent ecstasy-related cognitive deficits; the contribution of concomitant cannabis or other drug use to both brain imaging abnormalities and cognitive deficits; methodological shortcomings, such as failure to adequately match samples of ecstasy users and controls; the questionable relevance of animal models of MDMA-induced neurotoxicity to typical human patterns of ecstasy use [but see 72,92-94]; and the potential role of inherent pre-drug deficits in serotonergic systems, impulse control and executive cognitive function that may predispose to excessive use of drugs including ecstasy. Consequently, the actual neurotoxic potential of MDMA in humans remains a subject of much debate [25,37,95-100].

Importantly, many of the putative confounds of former clinical studies on the effects of MDMA in the serotonergic neurotransmitter system of abusers were carefully controlled in a recent study carried out by Kish and co-workers [101]. These authors using a magnetic resonance image for positron emission tomography image co-registration and structural analyses confirmed a caudorostral gradient of cortical SERT binding loss with occipital cortex most severely affected in ecstasy abusers in comparison to control subjects. The SERT binding loss was not related to structural changes or partial volume effect, use of other stimulant drugs, blood testosterone or oestradiol levels, major SERT gene promoter polymorphisms, gender, psychiatric status, or self-reported hyperthermia or tolerance. Furthermore, the ecstasy group, although ‘grossly behaviorally normal’, reported altered mood and demonstrated generally modest deficits on some tests of attention, executive function and memory associated with SERT decrease. The direct measurement of major brain 5-HT markers in human ecstasy users was also accomplished, for the first time, in a single post-mortem brain of a high-dose user [102]. The authors showed marked decreased levels of all 5-HT neuron markers: 5-HT and 5-HIAA content and protein concentrations of its rate limiting biosynthetic enzyme, tryptophan hydroxylase and of SERT [102]. These studies, therefore, appear to confirm the initial interpretation that serotonergic neurotransmission is subject to long lasting and subtle alterations following MDMA abuse in humans.

## 4. Interspecies Dose Scaling: Allometric *versus* Effect Scaling

To assess the potential risks to human health arising from chemical exposures such as MDMA, it is frequently necessary to rely on findings from controlled animal experiments. A major point of controversy relates to the relevance of MDMA doses administered to rats when compared to doses taken by humans [37]. The neurotoxic effects of MDMA in animals are dependent upon the number and size of doses given, the route of administration, the species receiving it, and the ambient temperature in which it is received [24,26]. Thus, MDMA regimens that produce 5-HT depletions in rats and non-human primates involve administration of single or multiple injections of 7.5-20 mg/kg or 5 mg/kg respectively, whereas the typical amount of MDMA taken by human users is 1-3 mg/kg.

Allometry is the measurement of the shape of an organism as it increases in size, and its principles have been used to extrapolate doses from one species to another. Allometric interspecies scaling is based on the assumption that there are anatomical, physiological, and biochemical similarities among animals, which can be described by mathematical models. It is now a well-established fact that many physiological processes and organ sizes exhibit a power-law relationship with the body weight of species. This relationship is the scientific basis of allometry. However, while some physiological, anatomical, and biochemical characteristics vary in a systematic manner, others do not. For example, (i) bioavailability and extent of binding to plasma proteins may be species-specific [103]; (ii) partition coefficients can also exhibit large differences across species [104]; (iii) qualitative differences between the human brain and those for other mammalian species (brain:body weight ratio and increased brain surface area due to folding in higher mammalian species) complicates the interspecies extrapolation for delivery to the brain [105]; (iv) allometric scaling yields poor predictions in humans for low clearance drugs eliminated primarily by the mixed function oxidase system [106]. Differences among animal species regarding other pharmacokinetic parameters such as drug absorption (bioavailability), variations in metabolic enzymes and their activities or the phenomenon of nonlinear kinetics are other factor that should be taken into account at the time of using the interspecies scaling to extrapolate toxic MDMA doses between animals and humans [reviewed by 28,107].

Due to the limitations of allometric scaling, other authors have proposed to use the effect scaling, as an alternative strategy for matching equivalent doses of MDMA in rats or non-human primates and humans [25, 108]. The principle behind interspecies effect scaling is that drug doses which induce identical effects (behavioral, physiological, neurochemical, etc.) in two different species may be considered equivalent, and therefore directly compared across these particular species, suggesting that the method of effect scaling can account for differences in ADME processes across species. Baumann *et al*. [25] compared the doses of MDMA capable of producing similar neurobiological effects in rats and humans (“effect scaling”). They concluded that there is no justification for using allometric interspecies scaling to adjust rats and human doses since MDMA produces comparable psychological effects at similar doses (∼1-2 mg/kg) in both species. Of notice, these findings contrast with other functional observations. Thus, a standard dose of MDMA (100 mg; 1.5 mg/kg p.o.) given to humans in a control setting increases oral temperature 0.6°C [109] and a similar rise is seen after a fourfold higher dose (5 mg/kg i.p.) administered to rats [110]; which, importantly, represents the rat/human dose ratio calculated by using the allometric interspecies scaling.

**Figure 2:**
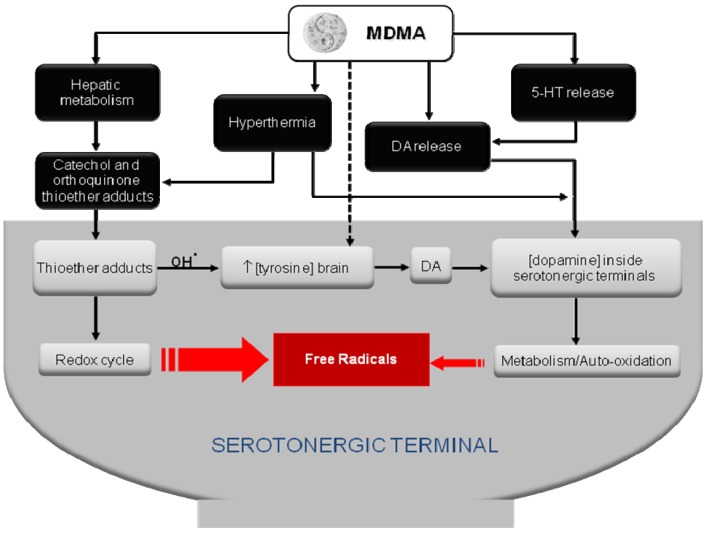
Proposed mechanisms underlying MDMA-induced serotonergic neurotoxicity. Toxic MDMA metabolites, increased tyrosine levels and dopamine metabolism act in concert inside the serotonergic terminals to promote oxidative stress and final terminal loss. Other factors, e.g. hyperthermia, by increasing MDMA metabolism, may contribute to MDMA-induced serotonergic deficits. Adapted from [115].

Which one of these two methods (allometric vs. effect scalling), if any, best predicts the dosage regimen that might result neurotoxic in humans based on neurotoxic studies carried out in rats or non-human primates awaits further studies. For instance, the effect scaling method predicts that the dose of 1.7 mg/kg of MDMA used by Vollenweider *et al*. [9] in healthy humans would be completely safe since repeated treatment in rats with behaviorally relevant doses of MDMA (1.5 mg/kg i.p.) does not cause any sign of long-term 5-HT deficits [25]. Similar findings were reported by Fantegrossi *et al*. [39], who found no significant differences in VMAT binding or tissue levels of 5-HT, 5-HIAA, dopamine or DOPAC in rhesus monkeys that self-administered MDMA over an 18-month period. By contrast, the allometric scaling method predicts the opposite because a dose of 1.7 mg/kg given to healthy human volunteers (or self-administered by ecstasy users) is in the range of those reported to be neurotoxic in rats and non-human primates [111-113]. Noteworthy, in this latter work Drs. McCann and Ricaurte [113] remarked that the principles of interspecies scaling do not apply when the metabolic disposition in particular animal species involves the formation of a unique neurotoxic metabolite. So far, it is still unclear which are the mechanisms underlying MDMA-induced long-term 5-HT deficits. Some authors have cautiously suggested that peak plasma concentration of MDMA (C_max_) is the pharmacokinetic parameter that best correlates with the degree of 5-HT loss both in rats and non-human primates [93,114]; however, other authors have proposed the metabolism of MDMA into toxic metabolite(s) as responsible for its neurotoxic effects (Figure 2), see [26,115] for further discussion.

As suggested elsewhere, the optimal strategy for relating MDMA data from animals to humans might be to compare pharmacokinetic parameters across species [28,107,116,117]. This is exactly what it was done in two recent studies in which the metabolic disposition of MDMA in laboratory animals (rats or squirrel monkeys) and humans was compared [116,117]. In the first study by Baumann and co-workers [116], rats received a low, but pharmacologically active, dose of 2 mg/kg or a 5-fold higher dose of MDMA via intraperitoneal, subcutaneous or oral routes. Neither dose given via any of the three routes of administration resulted in long-term depletions of 5-HT in the cortex or striatum of the rat. Of interest, the lower dose, given orally or intraperitoneally, yielded an MDMA C_max_ of approximately 46 and 200 ng/mL, respectively. Two conclusions can be withdrawn from these data: (i) the effect scaling method cannot be used to appropriately estimate exposure to MDMA in relation to dose administered; (ii) in rats, an MDMA C_max_ in the range of those reported in humans who receive 1.3 to 1.7 mg/kg orally in a control setting [118-122], which represents a typical recreational single dose [49], does not result in long-lasting 5-HT deficits. An interesting conclusion withdrawn from the second study by Mueller *et al*. [117] refers to the impossibility of producing identical exposures in squirrel monkeys and humans despite using the interspecies dose scaling due to big differences in *T*_1/2_ of MDMA between humans and monkeys. Thus, estimated equivalent doses through the use of interspecies dose scaling yields similar AUC values but not C_max_ values, being the opposite also true, that is, non-equivalent dosages produce comparable C_max_ values but different AUC values. Obviously, this also applies to the rat [116]. However, as discussed by Mueller *et al*. [117] in the case of MDMA the most important pharmacokinetic parameter to better predict the neurotoxicity induced by this amphetamine derivative awaits to be defined, mainly because we still do not know what is causing the long-lasting 5-HT deficits described in rats, monkeys or humans.

## 5. Can We Prevent the Long-Term 5-HT Deficits Caused by MDMA in Rats?

In the following section we will briefly summarize the different paradigms that have been shown to reverse the serotonergic deficits observed after MDMA administration. All the studies presented refer to experiments carried out in rats since these type of experiments have not been conducted in non-human primates.

### 5.1. Core Body Temperature and 5-HT Deficits

Many *in vivo* studies show the close relationship between hyperthermia and neurotoxicity engendered by MDMA [123-126]. In this regard, any pharmacological or non-pharmacological manipulations capable of preventing the acute hyperthermic response caused by MDMA also attenuate or reverse the serotonergic deficits induced by the drug.

Keeping animals at low ambient temperatures during MDMA administrations [123,126] or surgical treatments such as hypophysectomy or thyrophysectomy [127] prevent both the acute hyperthermia and the long-term deficits observed in the serotonergic system after its administration.

Additionally, many compounds, that were initially reported to be neuroprotective in rats, have been confirmed to afford protection because of an indirect effect by lowering body temperature. That is the case of some *N*-methyl-d-aspartic acid (NMDA) glutamate receptor antagonists (MK-801, CGS 19755, NBQX) [128]; the 5-HT2A receptor antagonist, ketanserin [129]; the γ-aminobutyric acid (GABAA) agonist, clomethiazole [130]; the dopamine synthesis inhibitor, α-methyl-*p*-tyrosine (AMPT) [129]; the anesthetic pentobarbitone [131] and the glucose transport inhibitor 2-deoxy-d-glucose [62]. In all these cases, when the core body temperature of rats was kept elevated, the neuroprotective effect of these drugs was lost. Of interest, the converse also occurs; any manipulation known to potentiate MDMA-induced hyperthermia, such as small increases in ambient temperature [124,132,133] or caffeine administration [134] enhance the serotonergic deficits.

### 5.2. Inhibition of ROS Formation

Several studies suggest the involvement of oxidative stress in the mechanism of MDMA neurotoxicity. For instance, the administration of free radical scavengers and antioxidants such as phenylbutylnitrone, ascorbate and α-lipoic acid attenuates the serotonergic deficits induced by the drug [135-137]. Supporting these evidences is the fact that enhancing neuronal anti-oxidative defense by acetyl-L-carnitine prevents the decrease of 5-HT levels in several regions of the rat brain after MDMA administration [138]. In addition, the antioxidant and glutathione precursor *N*-acetylcysteine also prevented *in vitro* thioether MDMA metabolite-induced neuronal death [139].

### 5.3. Dopamine and Its Metabolism by MAO-B

It had been proposed that one source of free radicals observed after MDMA administration is the metabolism of dopamine by monoamine oxidase-B (MAO-B) to produce H_2_O_2_ that is reduced by iron to produce hydroxyl radicals and a subsequent terminal degeneration [140-143]. This suggestion is supported by the fact that MDMA elicits dopamine release [144] and the dopamine precursor l-3,4-dihydroxyphenylalanine (DOPA) exacerbates MDMA-induced 5-HT depletions in the rat [145]. Conversely, inhibition of dopamine synthesis and blockade of its uptake by mazindol attenuates MDMA-induced long-term depletion of brain 5-HT content [146,147]. Moreover, inhibition of MAO-B, either by pharmacological agents (l-deprenyl also termed selegiline) or antisense oligonucleotide targeted at MAO-B, was shown to be protective [140,141,148,149]. Importantly, a recent study has shown that MAO-A inhibition by clorgyline had no protective effect on MDMA-induced toxicity but the opposite [150]. The concomitant use of MAO-A inhibitors and MDMA is counter indicated because of the resulting severe synergic toxicity.

It should also be noted, however, that rats depleted of vesicular and cytoplasmic dopamine stores by means of previous treatment with reserpine and α-methyl-*p*-tyrosine (AMPT) showed no deficits of 5-HT after MDMA. Importantly, these animals did not turned hyperthermic but hypothermic and when this effect was averted by raising ambient temperature, the 5-HT neuroprotective effects of reserpine and AMPT were no longer apparent, suggesting that dopamine per se is not essential for the expression of MDMA-induced 5-HT neurotoxicity [125].

### 5.4. Inhibition of Nitric Oxide Synthase and Peroxynitrite Formation

Darvesh *et al.* [151] demonstrated that the systemic administration of MDMA to rats significantly increased the formation of nitric oxide and the nitrotyrosine in the striatum. These results support the conclusion that nitrogen reactive species (formed by the reaction of NO with O2•− radicals) could also be involved in MDMA damage. So, systemic administration of the nitric oxide synthase (NOS) inhibitors *N*-nitro-l-arginine (l-NOARG) or *S*-methyl-l-thiocitrulline inhibits brain NOS activity and provides protection against MDMA-induced indole depletion without attenuating the acute hyperthermia induced by this amphetamine analog [151,152]. Of interest, some *in vitro* studies have also demonstrated that the non-selective NOS inhibitor, *N*-ω-nitro-l-arginine [153] and specific inhibitors of the inducible and neuronal nitric oxide synthase (NOS) [154] partially prevented MDMA neurotoxicity, further suggesting the involvement of reactive nitrogen species in the toxic effect.

### 5.5. Inhibition of SERT-Mediated Transport

The involvement of the 5-HT transporter (SERT) in the long-term effects induced by MDMA has been evidenced by many studies. Thus, the 5-HT uptake inhibitor fluoxetine given up to seven days before, at the time or up to six hours after MDMA, prevents both the acute increase in hydroxyl radical formation which follows MDMA [149] and the long-lasting serotonergic deficits without interfering with MDMA-induced hyperthermia [30, 55, 132,156]. This neuroprotective effect might be due to the blockade of the entry of a toxic metabolite of MDMA into the 5-HT nerve terminal [156]. However, taking into account the studies suggesting the involvement of MDMA metabolism in its neurotoxicity [126,133] and the fact that fluoxetine is a potent enzymatic inhibitor, the protection afforded by the SERT inhibitor may also be a result of a pharmacokinetic interaction between fluoxetine and MDMA [157].

### 5.6. Pharmacological Preconditioning

Recent studies have reported that intermittent administration of MDMA to adolescent and adult rats resulted in tolerance to subsequent 5-HT-depleting regimen of the drug, as well as to the long-term reduction in SERT immunoreactivity [158-160]. Noteworthy this neuroprotective effect was independent of any alteration in MDMA pharmacokinetics or MDMA-induced hyperthermia. In general, the neuroprotective effects of prior intermittent MDMA exposure can be considered an example of “preconditioning,” a phenomenon whereby low doses or brief exposures to noxious insults protect the brain and other tissues from future insults [161,162].

Additionally, Puerta *et al*. [163] have recently shown that sildenafil, given shortly before MDMA, also prevented the long-term 5-HT deficits caused by MDMA in rats by a preconditioning-like mechanism. Of interest, minoxidil also afforded protection against MDMA-induced 5-HT depletion by a similar mechanism [164] including the activation of ATP-sensitive potassium (KATP) channels, an essential initiator of preconditioning in different models [165-169]. Moreover, 3-nitropropionic acid, given 24 h before a toxic MDMA treatment, completely prevented 5-HT depletions by increasing NO production [170]; which is also involved in various preconditioning paradigms [171,172]. In all these studies, the neuroprotective effect was independent of any alteration in the hyperthermia induced by MDMA.

Thus, pharmacological preconditioning is also a valid strategy to prevent the 5-HT depletions caused by MDMA. Although the mechanisms underlying this protective effect remain to be determined, attenuation of ROS production after development of preconditioning during lethal challenges is thought to be one of the major end effectors in this process [173-175].

### 5.7. 5-HT Precursors

Treatment of rats with 5-HT precursors, tryptophan or 5-hydroxytryptophan, was shown to attenuate MDMA-induced serotonergic deficits as measured by [^3^H]-paroxetine binding and 5-HT content in the striatum, hippocampus, and frontal cortex of the rat brain [176]. These authors suggested that 5-HT depleted terminals are more vulnerable to the toxic effects of MDMA, and so, 5-HT precursors would protect 5-HT terminals by replenishing the vesicular stores.

## 6. Neuroadaptation *vs*. Neurotoxicity

During the last few years, several studies have questioned the neurotoxic potential of MDMA to 5-HT terminals. Such conclusions were based on results from western blot studies of the SERT protein showing no change in SERT protein abundance despite large decreases of 5-HT concentrations and [^3^H]paroxetine binding after, what up to that date, had been considered a neurotoxic MDMA treatment in rats [25,177,178]. However, results of western blot studies indicate that the 50 kDa band previously thought to correspond to the SERT protein [178] does not exhibit the known relative regional distribution of brain SERT, is resistant to the well established 5-HT neurotoxicant 5,7-dihydroxytryptamine, and is present in SERT-KO animals. It appears, therefore, that the failure of previous studies to demonstrate the loss of SERT protein by Western blot relies on the fact that the band corresponding to SERT protein was misidentified, probably due to the lack of positive and negative controls used by other authors [63,179]. Recent studies have shown a significant decreased abundance of a ∼65 kDa band, corresponding to the SERT protein, in MDMA-treated rats [64,159,179,180], further supporting the conclusions reached by the initial study carried out by Xie and co-workers [63].

The previously reported studies showing no loss of the SERT protein band after MDMA, is not the only argument some authors put forward to question the 5-HT neurotoxic potential of MDMA. Reactive astrogliosis defined as an increase in the number and size of astrocytes can occur as a result of numerous acute or chronic stresses compromising brain homeostasis. Glial fibrillary acidic protein (GFAP) is the major protein constituent of astroglial intermediate filaments and has been used as a marker to detect neuronal degeneration [181]. There are few studies reporting the use of this marker to examine MDMA-induced damage and most of them reported no changes in GFAP expression after several treatment regimens with MDMA known to deplete central 5-HT concentrations [177,178,181,182]. This was further supported by the lack of an MDMA mediated effect on markers of microglial activation such as heat shock protein expression and peripheral benzodiazepine receptor binding [177,178,182]. These and other findings continue to raise doubts among some investigators as to whether MDMA “serotonergic neurotoxicity” involves distal axotomy or alternatively a long-lasting downregulation of 5-HT synthesis and SERT expression by the serotonergic neurons. It should be noted however, that besides the study by Aguirre *et al*. [137], at least two other studies have documented increases in GFAP expression after MDMA although at three but not seven days after MDMA [183] or after a very high dose of the drug [184]. Of interest, data from different studies using 5,7-DHT are also not consistent, with some studies revealing increased GFAP expression after neurotoxic regimens [177], whereas others report similar findings in old but not in young animals [185] or no increase at all [186-188]. It has been suggested that a lack of GFAP expression increase may be due to an insufficiently strong signal after 5-HT neuronal degeneration [186]; that the use of this parameter for detecting degeneration of serotonergic terminals may have limitations [63] or, alternatively, that changes in GFAP expression are transient and can only be detected shortly after neuronal injury caused by MDMA [183].

In a recent study by Wang *et al*. [189], a group of rats was treated with a neurotoxic dosage regimen of MDMA and two weeks later these same animals received an injection of the 5-HT precursor 5-hydroxytryptophan. Interestingly, 5-HT levels in the brain of MDMA-pretreated rats were close to those of control animals. Because SERT or TPH activity are not necessary for the conversion of 5-hydroxytryptophan into 5-HT these authors interpreted these data as a proof of concept for the integrity of 5-HT terminals. Accordingly, they suggested that MDMA causes lasting neuroadaptative changes in 5-HT neurons rather than 5-HT terminal loss. Later on, a different report showed that a “neurotoxic” dosage regimen of MDMA in rats did not affect VMAT-2 protein expression in the hippocampus despite producing large reductions in SERT levels [64]. This scenario is not consistent with the idea of MDMA being neurotoxic to 5-HT terminals in rats but it rather supports the neuroregulatory hypothesis proposed by Wang *et al*. [189]. The study of Wang *et al*. [189] also evinced the difficulty in determining the effects of MDMA when measuring endpoints such as 5-HT content, SERT protein expression, binding or function. These measures are all indirect and subject to regulations. For instance, it is well known that MDMA inhibits the activity [190] and abundance of tryptophan hydroxylase [191] at least two weeks after MDMA. Because tryptophan hydroxylase is the rate-limiting enzyme for 5-HT synthesis, it is likely that decreases in this enzyme restrict the extent of 5-HT production, thus reducing the levels of this neurotransmitter regardless of whether or not axonal damage has occurred. In this same line, SERT binding is also subject to various regulatory processes, and changes in SERT binding levels cannot, as a single line of evidence, connote the loss of 5-HT terminals. For example, chronic treatment of rats with 5-HT selective reuptake inhibitors (SSRIs) leads to a marked loss of SERT binding and function comparable to MDMA [192], although, such changes are transient and not long-lasting like those produced by MDMA. Further support for the above contention comes from two recent studies. In the first report, Kivell and co-workers [193] showed that MDMA causes a redistribution of SERT from the cell surface to intracellular vesicles. These authors therefore suggested that the loss of SERT from the cell surface upon acute exposure to MDMA might contribute to the decreased SERT function seen in rats exposed to MDMA. However we should be cautious at the time of interpreting these latter results, since this is expected to be an acute effect of MDMA and cannot account for the very long-lasting reductions reported in rats, non-human primates or ecstasy users. In the second study, Biezonski and Meyer [64] reported a profound down-regulation of SERT gene expression accompanied by a significant reduction in the expression of the VMAT-2 gene in the dorsal and median raphe nuclei of rats two weeks after a high dosage regimen of MDMA. The authors indicated that decreased protein levels liable to regulation (e.g. SERT or VMAT-2 seen after MDMA) do not necessarily reflect neurodegeneration. While this is true and could explain the loss of SERT protein found after MDMA in different animal species, the fact is that the opposite is not less certain. Therefore, a down-regulation of SERT protein expression does not preclude the existence of 5-HT terminal degeneration. Another unsolved question relates to the duration of the effects reported by Biezonski and Meyer [64] and later confirmed by Kirilly [194], since SERT mRNA was found to be below control levels by two and three weeks after MDMA, respectively, while the effects of MDMA last between 6-12 months in rats [195] and beyond seven years in non-human primates [56]. Moreover, studies in non-human primates have shown that, over time, there is regrowth of ascending serotonin axonal projections after MDMA-induced injury but that a normal innervation pattern is not restored [71].

Wang *et al*. [189] proposed three possible models for MDMA effects on the serotonergic system: (1) neurodegeneration, (2) neuroadaptation, and (3) a mixed model involving a significant loss of serotonergic nerve terminals along with adaptative changes in the remaining terminals. While the new findings reported by Biezonski and Meyer [64] and Bonkale and Austin [191] are consistent with the neuroadaptative model, it should be noted that they do not preclude the possibility that MDMA causes a partial degeneration of serotonergic nerve terminals. In this regard, it has been shown that MDMA administration does not lead to ultrastructural alteration in the serotonergic dorsal raphe cell bodies and in their proximal neurites but causes impairment in cortical serotonergic axons. In these, the main ultrastructural alteration is the destruction of microtubules although a smaller portion of these axons probably undergoes an irreversible damage [196]. These data support the clinical findings reported by Kish *et al*. [101,102] and also the mixed model of neurotoxicity suggested by Wang *et al*. [189]. It is also important to consider that the “neurodegeneration” hypothesis can account for all the findings reported in the scientific literature while the “modulation” hypothesis cannot, at this moment. More studies are, therefore, warranted.

Finally we would like to mention that the terms “neurotoxicity” and “neurodegeneration” in relation to amphetamines have been used interchangeably, when the former can occur without the latter. In this regard, the National Institute of Neurological Disorders and Stroke (NINDS) defines neurotoxicity as follows: “*Neurotoxicity occurs when the exposure to natural or manmade toxic substances (neurotoxicants) alters the normal activity of the nervous system. This can eventually disrupt or even kill neurons, key cells that transmit and process signals in the brain and other parts of the nervous system. Neurotoxicity can result from exposure to substances used in chemotherapy, radiation treatment, drug therapies, and organ transplants, as well as exposure to heavy metals such as lead and mercury, certain foods and food additives, pesticides, industrial and/or cleaning solvents, cosmetics, and some naturally occurring substances. Symptoms may appear immediately after exposure or be delayed. They may include limb weakness or numbness; loss of memory, vision, and/or intellect; headache; cognitive and behavioral problems; and sexual dysfunction. Individuals with certain disorders may be especially vulnerable to neurotoxicants*”.

As reviewed above, MDMA, at the very least, causes severe deficits in different markers of the serotonergic neurotransmitter system in rodents, non-human primates and humans [24,26,101,102] and causes long-lasting cognitive and behavioral problems [48,52,197] and therefore fulfills the definition of a neurotoxicant compound provided by the NINDS. Whether, it also causes neurodegeneration of 5-HT axons still awaits confirmation.

## References

[1] UNODCC (United Nations Office of Drug Control and Crime) (2008). World Drug Report.

[2] EMCDDA (2010). 2010 Annual report on the State of the Drugs Problem in Europe.

[3] Downing J. (1986). The psychological and physiological effects of MDMA on normal volunteers. J. Psychoact. Drugs.

[4] Greer G., Tolbert R. (1986). Subjective reports of the effects of MDMA in a clinical setting. J. Psychoactive Drugs.

[5] Liechti M.E., Baumann C., Gamma A., Vollenweider F.X. (2000). Acute psychological effects of 3,4-methylenedioxymethamphetamine (MDMA, ‘Ecstasy’) are attenuated by the serotonin uptake inhibitor citalopram. Neuropsychopharmacology.

[6] Liechti M.E., Saur M.R., Gamma A., Hell D., Vollenweider F.X. (2000). Psychological and physiological effects of MDMA (‘Ecstasy’) after pretreatment with the 5-HT(2) antagonist ketanserin in healthy humans. Neuropsychopharmacology.

[7] Liechti M.E., Vollenweider F.X. (2000). Acute psychological and physiological effects of MDMA (‘Ecstasy’) after haloperidol pretreatment in healthy humans. Eur. Neuropsychopharmacol..

[8] Liechti M.E., Vollenweider F.X. (2001). Which neuroreceptors mediate the subjective effects of MDMA in humans? A summary of mechanistic studies. Hum. Psychopharmacol..

[9] Vollenweider F.X., Gamma A., Liechti M., Huber T. (1998). Psychological and cardiovascular effects and short-term sequelae of MDMA (“ecstasy”) in MDMA-naive healthy volunteers. Neuropsychopharmacology.

[10] Nichols D.E. (1986). Differences between the mechanism of action of MDMA, MBDB, and the classic hallucinogens. Identification of a new therapeutic class: Entactogens. J. Psychoact. Drugs.

[11] Grinspoon L., Bakalar J.B. (1986). Can drugs be used to enhance the psychotherapeutic process?. Am. J. Psychother..

[12] Freudenmann R.W., Oxler F., Bernschneider-Reif S. (2006). The origin of MDMA (ecstasy) revisited: the true story reconstructed from the original documents. Addiction.

[13] Grob C.S., Bravo G.L., Walsh R.N., Liester M.B. (1992). The MDMA-neurotoxicity controversy: Implications for clinical research with novel psychoactive drugs. J. Nerv. Ment. Dis..

[14] Liester M.B., Grob C.S., Bravo G.L., Walsh R.N. (1992). Phenomenology and sequelae of 3,4-methylenedioxymethamphetamine use. J. Nerv. Ment. Dis..

[15] Strassman R.J. (1995). Hallucinogenic drugs in psychiatric research and treatment. Perspectives and prospects. J. Nerv. Ment. Dis..

[16] Johansen P.Ø., Krebs T.S. (2009). How could MDMA (ecstasy) help anxiety disorders? A neurobiological rationale. J. Psychopharmacol..

[17] Mithoefer M.C., Wagner M.T., Mithoefer A.T., Jerome L., Doblin R. (2011). The safety and efficacy of {+/-}3,4-methylenedioxymethamphetamine-assisted psychotherapy in subjects with chronic, treatment-resistant posttraumatic stress disorder: The first randomized controlled pilot study. J. Psychopharmacol..

[18] Schmidt C.J., Wu L., Lovenberg W. (1986). Methylenedioxymethamphetamine: A potentially neurotoxic amphetamine analogue. Eur. J. Pharmacol..

[19] Schmidt C.J. (1987). Neurotoxicity of the psychedelic amphetamine, methylenedioxymethamphetamine. J. Pharmacol. Exp. Ther..

[20] Battaglia G., Yeh S.Y., O'Hearn E., Molliver M.E., Kuhar M.J., De Souza E.B. (1987). 3,4-Methylenedioxymethamphetamine and 3,4-methylenedioxyamphetamine destroy serotonin terminals in rat brain: Quantification of neurodegeneration by measurement of [3H]paroxetine-labeled serotonin uptake sites. J. Pharmacol. Exp. Ther..

[21] Battaglia G., Yeh S.Y., De Souza E.B. (1988). MDMA-induced neurotoxicity: Parameters of degeneration and recovery of brain serotonin neurons. Pharmacol. Biochem. Behav..

[22] Ricaurte G.A., DeLanney L.E., Wiener S.G., Irwin I., Langston J.W. (1988). 5-Hydroxyindoleacetic acid in cerebrospinal fluid reflects serotonergic damage induced by 3,4-methylenedioxymethamphetamine in CNS of non-human primates. Brain Res..

[23] Steele T.D., McCann U.D., Ricaurte G.A. (1994). 3,4-Methylenedioxymethamphetamine (MDMA, “Ecstasy”): Pharmacology and toxicology in animals and humans. Addiction.

[24] Green A.R., Mechan A.O., Elliott J.M., O'Shea E., Colado M.I. (2003). The pharmacology and clinical pharmacology of 3,4-methylenedioxymethamphetamine (MDMA, “ecstasy”). Pharmacol. Rev..

[25] Baumann M.H., Wang X., Rothman R.B. (2007). 3,4-Methylenedioxymethamphetamine (MDMA) neurotoxicity in rats: A reappraisal of past and present findings. Psychopharmacology (Berl).

[26] Capela J.P., Carmo H., Remião F., Bastos M.L., Meisel A., Carvalho F. (2009). Molecular and cellular mechanisms of ecstasy-induced neurotoxicity: An overview. Mol. Neurobiol..

[27] Steinkellner T., Freissmuth M., Sitte H.H., Montgomery T. (2011). The ugly side of amphetamines: Short- and long-term toxicity of 3,4-methylenedioxymethamphetamine (MDMA, ‘Ecstasy’), methamphetamine and d-amphetamine. Biol. Chem..

[28] de La Torre R., Farré M. (2004). Neurotoxicity of MDMA (ecstasy): The limitations of scaling from animals to humans. Trends Pharmacol. Sci..

[29] Johnson M.P., Hoffman A.J., Nichols D.E. (1986). Effects of the enantiomers of MDA, MDMA and related analogues on [3H]serotonin and [3H]dopamine release from superfused rat brain slices. Eur. J. Pharmacol..

[30] Schmidt C.J., Levin J.A., Lovenberg W. (1987). *In vitro* and *in vivo* neurochemical effects of methylenedioxymethamphetamine on striatal monoaminergic systems in the rat brain. Biochem. Pharmacol..

[31] Berger U.V., Gu X.F., Azmitia E.C. (1992). The substituted amphetamines 3,4-methylenedioxymethamphetamine, methamphetamine, pchloroamphetamine and fenfluramine induce 5-hydroxytryptamine release via a common mechanism blocked by fluoxetine and cocaine. Eur. J. Pharmacol..

[32] Fitzgerald J.L., Reid J.J. (1993). Interactions of methylenedioxymethamphetamine with monoamine transmitter release mechanisms in rat brain slices. Naunyn. Schmiedebergs Arch. Pharmacol..

[33] Crespi D., Mennini T., Gobbi M. (1997). Carrier-dependent and Ca(2+)-dependent 5-HT and dopamine release induced by (+)-amphetamine, 3,4-methylendioxymethamphetamine, *p*-chloro-amphetamine and (+)-fenfluramine. Br. J. Pharmacol..

[34] Rothman R.B., Baumann M.H., Dersch C.M., Romero D.V., Rice K.C., Carroll F.I., Partilla J.S. (2001). Amphetamine-type central nervous system stimulants release norepinephrine more potently than they release dopamine and serotonin. Synapse.

[35] Gudelsky G.A., Yamamoto B.K. (2008). Actions of 3,4-methylenedioxymethamphetamine (MDMA) on cerebral dopaminergic, serotonergic and cholinergic neurons. Pharmacol. Biochem. Behav..

[36] Liechti M.E., Geyer M.A., Hell D., Vollenweider F.X. (2001). Effects of MDMA (ecstasy) on prepulse inhibition and habituation of startle in humans after pretreatment with citalopram, haloperidol, or ketanserin. Neuropsychopharmacology.

[37] Cole J.C., Sumnall H.R. (2003). Altered states: The clinical effects of Ecstasy. Pharmacol. Ther..

[38] Ramos M., Goñi-Allo B., Aguirre N. (2004). Studies on the role of dopamine D1 receptors in the development and expression of MDMA-induced behavioral sensitization in rats. Psychopharmacology (Berl).

[39] Fantegrossi W.E., Woolverton W.L., Kilbourn M., Sherman P., Yuan J., Hatzidimitriou G., Ricaurte G.A., Woods J.H., Winger G. (2004). Behavioral and neurochemical consequences of long-term intravenous self-administration of MDMA and its enantiomers by rhesus monkeys. Neuropsychopharmacology.

[40] de La Garza R., Fabrizio K.R., Gupta A. (2007). Relevance of rodent models of intravenous MDMA self-administration to human MDMA consumption patterns. Psychopharmacology (Berl).

[41] Schenk S. (2011). MDMA (“ecstasy”) abuse as an example of dopamine neuroplasticity. Neurosci. Biobehav. Rev..

[42] Degenhardt L., Bruno R., Topp L. (2010). Is ecstasy a drug of dependence?. Drug Alcohol Depend..

[43] Parrott A.C. (2005). Chronic tolerance to recreational MDMA (3,4-methylenedioxymethamphetamine) or ecstasy. J. Psychopharmacol..

[44] Banken J.A. (2004). Drug Abuse Trends among Youth in the United States. Ann. N.Y. Acad. Sci..

[45] Schifano F. (2004). A bitter pill. Overview of ecstasy (MDMA, MDA) related fatalities. Psychopharmacology (Berl).

[46] Galicia M., Nogué S., Sanjurjo E., Miró O. (2010). Visits to the emergency department due to ecstasy (MDMA) and amphetamine derivative consumption: Epidemiological, clinical and evolutional profile. Rev. Clin. Esp..

[47] Morgan M.J. (2000). Ecstasy (MDMA): a review of its possible persistent psychological effects. Psychopharmacology (Berl).

[48] Soar K., Turner J.J., Parrott A.C. (2001). Psychiatric disorders in Ecstasy (MDMA) users: a literature review focusing on personal predisposition and drug history. Hum. Psychopharmacol..

[49] Parrott A.C. (2002). Recreational ecstasy/MDMA, the serotonin syndrome, and serotonergic neurotoxicity. Pharmacol. Biochem. Behav..

[50] Parrott A.C. (2006). MDMA in humans: factors which affect the neuropsychobiological profiles of recreational ecstasy users, the integrative role of bioenergetic stress. J. Psychopharmacol..

[51] Kalechstein A.D., de La Garza R., Mahoney J.J., Fantegrossi W.E., Newton T.F. (2007). MDMA use and neurocognition: A meta-analytic review. Psychopharmacology (Berl).

[52] Zakzanis K.K., Campbell Z., Jovanovski D. (2007). The neuropsychology of ecstasy (MDMA) use: a quantitative review. Hum. Psychopharmacol..

[53] Karlsen S.N., Spigset O., Slordal L. (2008). The dark side of ecstasy: Neuropsychiatric symptoms after exposure to 3,4-methylenedioxymethamphetamine. Basic Clin. Pharmacol. Toxicol..

[54] Rogers G., Elston J., Garside R., Roome C., Taylor R., Younger P., Zawada A., Somerville M. (2009). The harmful health effects of recreational ecstasy: a systematic review of observational evidence. Health Technol Assess..

[55] Aguirre N., Ballaz S., Lasheras B., Del Río J. (1998). MDMA (“Ecstasy”) enhances 5-HT1A receptor density and 8-OH-DPAT-induced hypothermia: blockade by drugs preventing 5-hydroxytryptamine depletion. Eur. J. Pharmacol..

[56] Hatzidimitriou G., McCann U.D., Ricaurte G.A. (1999). Altered serotonin innervation patterns in the forebrain of monkeys treated with (±) 3,4-methylenedioxymethamphetamine seven years previously: factors influencing abnormal recovery. J. Neurosci..

[57] Hatzidimitriou G., Tsai E.H., McCann U.D., Ricaurte G.A. (2002). Altered prolactin response to M-chlorophenylpiperazine in monkeys previously treated with 3,4-methylenedioxymethamphetamine (MDMA) or fenfluramine. Synapse.

[58] McCann U.D., Szabo Z., Seckin E., Rosenblatt P., Mathews W.B., Ravert H.T., Dannals R.F., Ricaurte G.A. (2005). Quantitative PET studies of the serotonin transporter in MDMA users and controls using [11C]McN5652 and [11C]DASB. Neuropsychopharmacology.

[59] Stone D.M., Johnson M., Hanson G.R., Gibb J.W. (1989). Acute inactivation of tryptophan hydroxylase by amphetamine analogs involves the oxidation of sulfhydryl sites. Eur. J. Pharmacol..

[60] McCann U.D., Ridenour A., Shaham Y., Ricaurte G.A. (1994). Serotonin neurotoxicity after (+/-)3,4-methylenedioxymethamphetamine (MDMA; “ecstasy”): A Controlled Study in Humans. Neuropsychopharmacology.

[61] Aguirre N., Galbete J.L., Lasheras B., del Río J. (1995). Methylenedioxymethamphetamine induces opposite changes in central pre- and postsynaptic 5-HT1A receptors in rats. Eur. J. Pharmacol..

[62] Hervias I., Lasheras B., Aguirre N. (2000). 2-Deoxy-d-glucose prevents and nicotinamide potentiates 3,4-methylenedioxymethamphetamine- induced serotonin neurotoxicity. J. Neurochem..

[63] Xie T., Tong L., McLane M.W., Hatzidimitriou G., Yuan J., McCann U., Ricaurte G. (2006). Loss of serotonin transporter protein after MDMA and other ring-substituted amphetamines. Neuropsychopharmacology.

[64] Biezonski D.K., Meyer J.S. (2010). Effects of 3,4-methylenedioxymethamphetamine (MDMA) on serotonin transporter and vesicular monoamine transporter 2 protein and gene expression in rats: Implications for MDMA Neurotoxicity. J. Neurochem..

[65] McCann U.D., Eligulashvili V., Mertl M., Murphy D.L., Ricaurte G.A. (1999). Altered neuroendocrine and behavioral responses to m-chlorophenylpiperazine in 3,4-methylenedioxymethamphetamine (MDMA) users. Psychopharmacology (Berl).

[66] McCann U.D., Eligulashvili V., Ricaurte G.A. (2000). (±) 3,4-Methylenedioxymethamphetamine (‘Ecstasy’)-induced serotonin neurotoxicity: Clinical Studies. Neuropsychobiology.

[67] Taffe M.A., Davis S.A., Yuan J., Schroeder R., Hatzidimitriou G., Parsons L.H., Ricaurte G.A., Gold L.H. (2002). Cognitive performance of MDMA-treated rhesus monkeys: Sensitivity to Serotonergic Challenge. Neuropsychopharmacology.

[68] Andó R.D., Ádori C., Kirilly E., Molnár E., Kovács G.G., Ferrington L., Kelly P.A., Bagdy G. (2010). Acute SSRI-induced anxiogenic and brain metabolic effects are attenuated 6 months after initial MDMA-induced depletion. Behav. Brain Res..

[69] Jones K., Brennan K.A., Colussi-Mas J., Schenk S. (2010). Tolerance to 3,4-methylenedioxymethamphetamine is associated with impaired serotonin release. Addict. Biol..

[70] Ricaurte G.A., McCann U.D. (1992). Neurotoxic amphetamine analogues: Effects in monkeys and implications for humans. Ann. N. Y. Acad. Sci..

[71] Fischer C., Hatzidimitriou G., Wlos J., Katz J., Ricaurte G. (1995). Reorganization of ascending 5-HT axon projections in animals previously exposed to the recreational drug (+/-)3,4-methylenedioxymethamphetamine (MDMA, “ecstasy”). J. Neurosci..

[72] Kovács G.G., Andó R.D., Ádori C., Kirilly E., Benedek A., Palkovits M., Bagdy G. (2007). Single dose of MDMA causes extensive decrement of serotoninergic fibre density without blockage of the fast axonal transport in Dark Agouti rat brain and spinal cord. Neuropathol. Appl. Neurobiol..

[73] Commins D.L., Vosmer G., Virus R.M., Woolverton W.L., Schuster C.R., Seiden L.S. (1987). Biochemical and histological evidence that methylenedioxymethylamphetamine (MDMA) is toxic to neurons in the rat brain. J. Pharmacol. Exp. Ther..

[74] O'Hearn E., Battaglia G., De Souza E.B., Kuhar M.J., Molliver M.E. (1988). Methylenedioxyamphetamine (MDA) and methylenedioxymethamphetamine (MDMA) cause selective ablation of serotonergic axon terminals in forebrain: immunocytochemical evidence for neurotoxicity. J. Neurosci..

[75] Stone D.M., Hanson G.R., Gibb J.W. (1987). Differences in the central serotonergic effects of methylenedioxymethamphetamine (MDMA) in mice and rats. Neuropharmacology.

[76] Logan B.J., Laverty R., Sanderson W.D., Yee Y.B. (1988). Differences between rats and mice in MDMA (methylenedioxymethylamphetamine) neurotoxicity. Eur. J. Pharmacol..

[77] O'Callaghan J.P., Miller D.B. (1994). Neurotoxicity profiles of substituted amphetamines in the C57BL/6J mouse. J. Pharmacol. Exp. Ther..

[78] Granado N., Escobedo I., O'Shea E., Colado I., Moratalla R. (2008). Early loss of dopaminergic terminals in striosomes after MDMA administration to mice. Synapse.

[79] Granado N., O'Shea E., Bove J., Vila M., Colado M.I., Moratalla R. (2008). Persistent MDMA-induced dopaminergic neurotoxicity in the striatum and substantia nigra of mice. J. Neurochem..

[80] Ricaurte G.A., Finnegan K.T., Irwin I., Langston J.W. (1990). Aminergic metabolites in cerebrospinal fluid of humans previously exposed MDMA: Preliminary Observations. Ann. N. Y. Acad. Sci..

[81] Price L.H., Ricaurte G.A., Krystal J.H., Heninger G.R. (1989). Neuroendocrine and mood responses to intravenous l-tryptophan in 3,4-methylenedioxymethamphetamine (MDMA) users. Preliminary observations. Arch. Gen. Psychiatry.

[82] McCann U.D., Szabo Z., Scheffel U., Dannals R.F., Ricaurte G.A. (1998). Positron emission tomographic evidence of toxic effect of MDMA (“ecstasy”) on brain serotonin neurons in human beings. Lancet.

[83] McCann U.D., Szabo Z., Vranesic M., Palermo M., Mathews W.B., Ravert H.T., Dannals R.F., Ricaurte G.A. (2008). Positron emission tomographic studies of brain dopamine and serotonin transporters in abstinent (±)3,4-methylenedioxymethamphetamine (“ecstasy”) users: relationship to cognitive performance. Psychopharmacology (Berl).

[84] Bolla K.I., McCann U.D., Ricaurte G.A. (1998). Memory impairment in abstinent MDMA (“Ecstasy”) users. Neurology.

[85] Semple D.M., Ebmeier K.P., Glabus M.F., O'Carroll R.E., Johnstone E.C. (1999). Reduced *in vivo* binding to the serotonin transporter in the cerebral cortex of MDMA (‘ecstasy’) users. Br. J. Psychiatry..

[86] Gerra G., Zaimovic A., Ferri M., Zambelli U., Timpano M., Neri E., Marzocchi G.F., Delsignore R., Brambilla F. (2000). Longlasting effects of (±)3,4-methylenedioxymethamphetamine (ecstasy) on serotonin system function in humans. Biol. Psychiatry.

[87] Kish S.J., Furukawa Y., Ang L., Vorce S.P., Kalasinsky K.S. (2000). Striatal serotonin is depleted in brain of a human MDMA (ecstasy) user. Neurology.

[88] Buchert R., Obrocki J., Thomasius R., Vaterlein O., Petersen K., Jenicke L., Bohuslavizki K.H., Clausen M. (2001). Long-term effects of ‘ecstasy’ abuse on the human brain studied by FDG PET. Nucl. Med. Commun..

[89] Ricaurte G.A., McCann U.D., Szabo Z., Scheffel U. (2000). Toxicodynamics and long-term toxicity of the recreational drug, 3,4-methylenedioxymethamphetamine (MDMA, ‘Ecstasy’). Toxicol. Lett..

[90] Buchert R., Thomasius R., Nebeling B., Petersen K., Obrocki J., Jenicke L., Wilke F., Wartberg L., Zapletalova P., Clausen M. (2003). Long-term effects of ‘ecstasy’ use on serotonin transporters of the brain investigated by PET. J. Nucl. Med..

[91] de Win M.M., Jager G., Booij J., Reneman L., Schilt T., Lavini C., Olabarriaga S.D., den Heeten G.J., van den Brink W. (2008). Sustained effects of ecstasy on the human brain: a prospective neuroimaging study in novel users. Brain.

[92] Meyer J.S., Piper B.J., Vancollie V.E. (2008). Development and characterization of a novel animal model of intermittent MDMA (“Ecstasy”) exposure during adolescence. Ann. N. Y. Acad. Sci..

[93] Mechan A., Yuan J., Hatzidimitriou G., Irvine R.J., McCann U.D., Ricaurte G.A. (2006). Pharmacokinetic profile of single and repeated oral doses of MDMA in squirrel monkeys: relationship to lasting effects on brain serotonin neurons. Neuropsychopharmacology.

[94] Ádori C., Andó R.D., Szekeres M., Gutknecht L., Kovács G.G., Hunyady L., Lesch K.P., Bagdy G. (2011). Recovery and aging of serotonergic fibers after single and intermittent MDMA treatments in Dark Agouti rat. J. Comp. Neurol..

[95] Curran H.V. (2000). Is MDMA (‘Ecstasy’) neurotoxic in humans? An overview of evidence and of methodological problems in research. Neuropsychobiology.

[96] Kish S.J. (2002). How strong is the evidence that brain serotonin neurons are damaged in human users of ecstasy?. Pharmacol. Biochem. Behav..

[97] Schreckenberger M. (2006). “Ecstasy”-induced neurotoxicity: The contribution of functional brain imaging. Eur. J. Nucl. Med. Mol. Imaging.

[98] Gouzoulis-Mayfrank E., Daumann J. (2006). Neurotoxicity of methylenedioxyamphetamines (MDMA; ecstasy) in humans, how strong is the evidence for persistent brain damage?. Addiction.

[99] Lyvers M. (2006). Recreational ecstasy use and the neurotoxic potential of MDMA: Current status of the controversy and methodological issues. Drug Alcohol Rev..

[100] Krebs T.S., Johansen P.Ø., Jerome L., Halpern J.H. (2009). Importance of psychiatric confounding in non-randomized studies of heavy ecstasy users. Psychol. Med..

[101] Kish S.J., Lerch J., Furukawa Y., Tong J., McCluskey T., Wilkins D., Houle S., Meyer J., Mundo E., Wilson A.A. (2010). Decreased cerebral cortical serotonin transporter binding in ecstasy users: A positron emission tomography/[11C]DASB and structural brain imaging study. Brain.

[102] Kish S.J., Fitzmaurice P.S., Chang L.J., Furukawa Y., Tong J. (2010). Low striatal serotonin transporter protein in human polydrug MDMA (Ecstasy) user: a case study. J. Psychopharmacol..

[103] Lin J.H. (1998). Applications and limitations of interspecies scaling and *in vitro* extrapolation in pharmacokinetics. Drug Metab. Dispos..

[104] Niazi S., Chiou W.L. (1975). Fluorocarbon aerosol propellants VI: Interspecies differences in solubilities in blood and plasma and their possible implications in toxicity studies. J. Pharm. Sci..

[105] Dedrick R.L. (1986). Interspecies scaling of regional drug delivery. J. Pharm. Sci..

[106] Ings R.M.J. (1990). Interspecies scaling and comparisons in drug development and toxicokinetics. Xenobiotica.

[107] Green A.R., Gabrielsson J., Marsden C.A., Fone K.C. (2009). MDMA: On the translation from rodent to human dosing. Psychopharmacology (Berl).

[108] Fantegrossi W.E. (2007). Reinforcing effects of methylenedioxy amphetamine congeners in rhesus monkeys: Are intravenous self-administration experiments relevant to MDMA neurotoxicity?. Psychopharmacology (Berl).

[109] Farré M., Abanades S., Roset P.N., Peiro A.M., Torrens M., O'Mathuna B., Segura M., de La Torre R. (2007). Pharmacological interaction between 3,4-methylenedioxymethamphetamine (ecstasy) and paroxetine: Pharmacological effects and pharmacokinetics. J. Pharmacol. Exp. Ther..

[110] Colado M.I., Williams J.L., Green A.R. (1995). The hyperthermic and neurotoxic effects of ‘Ecstasy’ (MDMA) and 3,4 methylenedioxyamphetamine (MDA) in the Dark Agouti (DA) rat, a model of the CYP2D6 poor metabolizer phenotype. Br. J. Pharmacol..

[111] Gijsman H.J., Verkes R.J., van Gerven J.M., Cohen A.F. (1999). MDMA study. Neuropsychopharmacology.

[112] Lieberman J.A., Aghajanian G.K. (1999). Caveat emptor: researcher beware. Neuropsychopharmacology.

[113] McCann U.D., Ricaurte G.A. (2001). Caveat emptor: editors beware. Neuropsychopharmacology.

[114] Mueller M., Yuan J., Felim A., Neudörffer A., Peters F.T., Maurer H.H., McCann U.D., Largeron M., Ricaurte G.A. (2009). Further studies on the role of metabolites in (±)-3,4-methylenedioxymethamphetamine-induced serotonergic neurotoxicity. Drug Metab. Dispos..

[115] Puerta E., Hervias I., Aguirre N. (2009). On the mechanisms underlying 3,4-methylenedioxymethamphetamine toxicity: The dilemma of the chicken and the egg. Neuropsychobiology.

[116] Baumann M.H., Zolkowska D., Kim I., Scheidweiler K.B., Rothman R.B., Huestis M.A. (2009). Effects of dose and route of administration on pharmacokinetics of (+ or -)-3,4-methylenedioxymethamphetamine in the rat. Drug Metab. Dispos..

[117] Mueller M., Kolbrich E.A., Peters F.T., Maurer H.H., McCann U.D., Huestis M.A., Ricaurte G.A. (2009). Direct comparison of (±) 3,4-methylenedioxymethamphetamine (“ecstasy”) disposition and metabolism in squirrel monkeys and humans. Ther. Drug Monit..

[118] Mas M., Farré M., de La Torre R., Roset P.N., Ortuño J., Segura J., Camí J. (1999). Cardiovascular and neuroendocrine effects and pharmacokinetics of 3,4-methylenedioxymethamphetamine in humans. J. Pharmacol. Exp. Ther..

[119] de La Torre R., Farré M., Ortuño J., Mas M., Brenneisen R., Roset P.N., Segura J., Camí J. (2000). Non-linear pharmacokinetics of MDMA (‘ecstasy’) in humans. Br. J. Clin. Pharmacol..

[120] Segura M., Ortuño J., Farré M., McLure J.A., Pujadas M., Pizarro N., Llebaria A., Joglar J., Roset P.N., Segura J., de La Torre R. (2001). 3,4-Dihydroxymethamphetamine (HHMA). A major *in vivo* 3,4-methylenedioxymethamphetamine (MDMA) metabolite in humans. Chem. Res. Toxicol..

[121] Hernández-López C., Farré M., Roset P.N., Menoyo E., Pizarro N., Ortuño J., Torrens M., Camí J., de La Torre R. (2002). 3,4-Methylenedioxymethamphetamine (ecstasy) and alcohol interactions in humans: psychomotor performance, subjective effects, and pharmacokinetics. J. Pharmacol. Exp. Ther..

[122] Kolbrich E.A., Goodwin R.S., Gorelick D.A., Hayes R.J., Stein E.A., Huestis M.A. (2008). Physiological and subjective responses to controlled oral 3,4-methylenedioxymethamphetamine administration. J. Clin. Psychopharmacol..

[123] Broening H.W., Bowyer J.F., Slikker W. (1995). Age- dependent sensitivity of rats to the long-term effects of the serotonergic neurotoxicant (±)3,4-methylenedioxymethamphetamine (MDMA) correlates with the magnitude of the MDMA-induced thermal response. J. Pharmacol. Exp. Ther..

[124] Malberg J.E., Seiden L.S. (1998). Small changes in ambient temperature cause large changes in 3,4-methylenedioxymethamphetamine (MDMA)-induced serotonin neurotoxicity and core body temperature in the rat. J. Neurosci..

[125] Yuan J., Cord B.J., McCann U.D., Callahan B.T., Ricaurte G.A. (2002). Effect of depleting vesicular and cytoplasmic dopamine on methylenedioxymethamphetamine neurotoxicity. J. Neurochem..

[126] Goñi-Allo B., O Mathúna B., Segura M., Puerta E., Lasheras B., de La Torre R., Aguirre N. (2008). The relationship between core body temperature and 3,4-methylenedioxymethamphetamine metabolism in rats: implications for neurotoxicity. Psychopharmacology (Berl).

[127] Sprague J.E., Banks M.L., Cook V.J., Mills E.M. (2003). Hypothalamic-pituitary-thyroid axis and sympathetic nervous system involvement in hyperthermia induced by 3,4-methylenedioxymethamphetamine (Ecstasy). J. Pharmacol. Exp. Ther..

[128] Farfel G.M., Seiden L.S. (1995). Role of hypothermia in the mechanism of protection against serotonergic toxicity. Experi- ments using 3,4-methylenedioxymethamphetamine, dizocilpine, CGS 19755 and NBQX. J. Pharmacol. Exp. Ther..

[129] Malberg J.E., Sabol K,E., Seiden L.S. (1996). Co-administration of MDMA with drugs that protect against MDMA neurotoxicity produces different effects on body temperature in the rat. J. Pharmacol. Exp. Ther..

[130] Colado M.I., Granados R., O'Shea E., Esteban B., Green A.R. (1998). Role of hyperthermia in the protective action of clomethiazole against MDMA (‘ecstasy’)-induced neurodegeneration, comparison with the novel NMDA channel blocker AR-R15896AR. Br. J. Pharmacol..

[131] Colado M.I., Esteban B., O'Shea E., Granados R., Green A.R. (1999). Studies on the neuroprotective effect of pentobarbitone on MDMA-induced neurodegeneration. Psychopharmacology.

[132] Goñi-Allo B., Puerta E., Hervias I., Di Palma R., Ramos M., Lasheras B., Aguirre N. (2007). Studies on the mechanisms underlying amiloride enhancement of 3,4-methylenedioxymethamphetamine-induced serotonin depletion in rats. Eur. J. Pharmacol..

[133] Goñi-Allo B., Puerta E., Mathúna B.O., Hervias I., Lasheras B., de La Torre R., Aguirre N. (2008). On the role of tyrosine and peripheral metabolism in 3,4-methylenedioxymethamphetamine-induced serotonin neurotoxicity in rats. Neuropharmacology.

[134] McNamara R., Kerans A., O'Neill B., Harkin A. (2006). Caffeine promotes hyperthermia and serotonergic loss following co- administration of the substituted amphetamines, MDMA (“ecstasy”) and MDA (“love”). Neuropharmacology.

[135] Colado M.I., O'Shea E., Granados R., Murray T.K., Green A.R. (1997). *In vivo* evidence for free radical involvement in the degeneration of rat brain 5-HT following administration of MDMA (‘ecstasy’) and *p*-chloroamphetamine but not the degeneration following fenfluramine. Br. J. Pharmacol..

[136] Gudelsky G.A. (1996). Effect of ascorbate and cysteine on the 3,4-methylenedioxymethamphetamine-induced depletion of brain serotonin. J. Neural. Transm..

[137] Aguirre N., Barrionuevo M., Ramírez M.J., Del Río J., Lasheras B. (1999). Alpha-lipoic acid prevents 3,4-methylenedioxymethamphetamine (MDMA)-induced neurotoxicity. Neuroreport.

[138] Alves E., Binienda Z., Carvalho F., Alves C.J., Fernandes E., de Lourdes Bastos M., Tavares M.A., Summavielle T. (2009). Acetyl-l-carnitine provides effective *in vivo* neuroprotection over 3,4-methylenedioximethamphetamine-induced mitochondrial neurotoxicity in the adolescent rat brain. Neuroscience.

[139] Capela J.P., Macedo C., Branco P.S., Ferreira L.M., Lobo A.M., Fernandes E., Remião F., Bastos M.L., Dirnagl U., Meisel A., Carvalho F. (2007). Neurotoxicity mechanisms of thioether ecstasy metabolites. Neuroscience.

[140] Sprague J.E., Nichols D.E. (1995). Inhibition of MAO-B protects against MDMA-induced neurotoxicity in the striatum. Psychopharmacology.

[141] Sprague J.E., Nichols D.E. (1995). The monoamine oxidase-B inhibitor l-deprenyl protects against 3,4-methylenedioxymethamphetamine- induced lipid peroxidation and long-term serotonergic deficits. J. Pharmacol. Exp. Ther..

[142] Sprague J.E., Everman S.L., Nichols D.E. (1998). An integrated hypothesis for the serotonergic axonal loss induced by 3,4-methylenedioxymethamphetamine. Neurotoxicology.

[143] Hrometz S.L., Brown A.W., Nichols D.E., Sprague J.E. (2004). 3,4-methylenedioxymethamphetamine (MDMA, ecstasy)-mediated production of hydrogen peroxide in an *in vitro* model: The role of dopamine, the serotonin-reuptake transporter, and monoamine oxidase-B. Neurosci. Lett..

[144] Yamamoto B.K., Spanos L.J. (1988). The acute effects of methylenedioxymethamphetamine on dopamine release in the awake-behaving rat. Eur. J. Pharmacol..

[145] Schmidt C.J., Black C.K., Taylor V.L. (1991). l-DOPA potentiation of the serotonergic deficits due to a single administration of 3,4-methylenedioxymethamphetamine, p-chloroamphetamine or methamphetamine to rats. Eur. J. Pharmacol..

[146] Schmidt C.J., Taylor V.L., Abbate G.M., Nieduzak T.R. (1991). 5-HT2 antagonists stereoselectively prevent the neurotoxicity of 3,4-methylenedioxymethamphetamine by blocking the acute stimulation of dopamine synthesis: reversal by l-DOPA. J. Pharmacol. Exp. Ther..

[147] Shankaran M., Yamamoto B.K., Gudelsky G.A. (1999). Mazindol attenuates the 3,4-methylenedioxymethamphetamine-induced formation of hydroxyl radicals and long-term depletion of serotonin in the striatum. J. Neurochem..

[148] Falk E.M., Cook V.J., Nichols D.E., Sprague J.E. (2002). An antisense oligonucleotide targeted at MAO-B attenuates rat striatal serotonergic neurotoxicity induced by MDMA. Pharmacol. Biochem. Behav..

[149] Alves E., Summavielle T., Alves C.J., Gomes-da-Silva J., Barata J.C., Fernandes E., Bastos M.L., Tavares M.A., Carvalho F. (2007). Monoamine oxidase-B mediates ecstasy-induced neurotoxic effects to adolescent rat brain mitochondria. J. Neurosci..

[150] Alves E., Summavielle T., Alves C.J., Custódio J.B., Fernandes E., de Lourdes Bastos M., Tavares M.A., Carvalho F. (2009). Ecstasy-induced oxidative stress to adolescent rat brain mitochondria *in vivo*: Influence of Monoamine Oxidase Type A. Addict Biol..

[151] Darvesh A.S., Yamamoto B.K., Gudelsky G.A. (2005). Evidence for the involvement of nitric oxide in 3,4-methylenedioxymethamphetamine-induced serotonin depletion in the rat brain. J. Pharmacol. Exp. Ther..

[152] Zheng Y., Laverty R. (1998). Role of brain nitric oxide in 3,4-methylenedioxymethamphetamine (MDMA)-induced neurotoxicity in rats. Brain Res..

[153] Capela J.P., Ruscher K., Lautenschlager M., Freyer D., Dirnagl U., Gaio A.R., Bastos M.L., Meisel A., Carvalho F. (2006). Ecstasy induced cell death in cortical neuronal cultures is serotonin 2a receptor-dependent and potentiated under hyperthermia. Neuroscience.

[154] Capela J.P., Fernandes E., Remiao F., Bastos M.L., Meisel A., Carvalho F. (2007). Ecstasy induces apoptosis via 5-HT2a-receptor stimulation in cortical neurons. Neurotoxicology.

[155] Shankaran M., Yamamoto B.K., Gudelsky G.A. (1999). Involvement of the serotonin transporter in the formation of hydroxyl radicals induced by 3,4-methylenedioxymethamphetamine. Eur. J. Pharmacol..

[156] Sanchez V., Camarero J., Esteban B., Peter M.J., Green A.R., Colado M.I. (2001). The mechanisms involved in the long-lasting neuroprotective effect of fluoxetine against MDMA (‘ecstasy’)-induced degeneration of 5-HT nerve endings in rat brain. Br. J. Pharmacol..

[157] Upreti V.V., Eddington N.D. (2008). Fluoxetine pretreatment effects pharmacokinetics of 3,4-methylenedioxymethamphetamine (MDMA, “ecstasy”) in rat. J. Pharm. Sci..

[158] Piper B.J., Vu H.L., Safain M.G., Oliver A.J., Meyer J.S. (2006). Repeated adolescent 3,4-methylenedioxymethamphetamine (MDMA) exposure in rats attenuates the effects of a subsequent challenge with MDMA or a 5-hydroxytryptamine(1A) receptor agonist. J. Pharmacol. Exp. Ther..

[159] Bhide N.S., Lipton J.W., Cunningham J.I., Yamamoto B.K., Gudelsky G.A. (2009). Repeated exposure to MDMA provides neuroprotection against subsequent MDMA-induced serotonin depletion in brain. Brain Res..

[160] Piper B.J., Ali S.F., Daniels L.G., Meyer J.S. (2010). Repeated intermittent methylenedioxymethamphetamine exposure protects against the behavioral and neurotoxic, but not hyperthermic, effects of an MDMA binge in adult rats. Synapse.

[161] Kirino T. (2002). Ischemic tolerance. J. Cereb. Blood Flow Metab..

[162] Sharp F.R., Ran R., Lu A., Tang Y., Strauss K.I., Glass T., Ardizzone T., Bernaudin M. (2004). Hypoxic preconditioning protects against ischemic brain injury. NeuroRx..

[163] Puerta E., Hervias I., Goñi-Allo B., Lasheras B., Jordan J., Aguirre N. (2009). Phosphodiesterase 5 inhibitors prevent 3,4-methylenedioxymethamphetamine-induced 5-HT deficits in the rat. J. Neurochem..

[164] Goñi-Allo B., Puerta E., Ramos M., Lasheras B., Jordán J., Aguirre N. (2008). Minoxidil prevents 3,4-methylenedioxymethamphetamine-induced serotonin depletions: role of mitochondrial ATP-sensitive potassium channels, Akt and ERK. J. Neurochem..

[165] Heurteaux C., Lauritzen I., Widmann C., Lazdunski M. (1995). Essential role of adenosine, adenosine A1 receptors, and ATPsensitive K+ channels in cerebral ischemic preconditioning. Proc. Natl. Acad. Sci. USA.

[166] Blondeau N., Plamondon H., Richelme C., Heurteaux C., Lazdunski M. (2000). K(ATP) channel openers, adenosine agonists and epileptic preconditioning are stress signals inducing hippocampal neuroprotection. Neuroscience.

[167] Tai K.K., Truong D.D. (2002). Activation of adenosine triphosphate-sensitive potassium channels confers protection against rotenone-induced cell death: therapeutic implications for Parkinson's disease. J. Neurosci. Res..

[168] O'Rourke B. (2000). Myocardial K(ATP) channels in preconditioning. Circ. Res..

[169] Busija D.W., Gaspar T., Domoki F., Katakam P.V., Bari F. (2008). Mitochondrial-mediated suppression of ROS production upon exposure of neurons to lethal stress: mitochondrial targeted preconditioning. Adv. Drug Deliv. Rev..

[170] Puerta E., Pastor F., Dvoracek J., de Saavedra M.D., Goñi-Allo B., Jordán J., Hervias I., Aguirre N. (2010). Delayed pre-conditioning by 3-nitropropionic acid prevents 3,4-methylenedioxymetamphetamine-induced 5-HT deficits. J. Neurochem..

[171] Huang P.L. (2004). Nitric oxide and cerebral ischemic preconditioning. Cell Calcium.

[172] Pignataro G., Scorziello A., Di Renzo G., Annunziato L. (2009). Post-ischemic brain damage: effect of ischemic preconditioning and postconditioning and identification of potential candidates for stroke therapy. FEBS J..

[173] Teshima Y., Akao M., Li R.A., Chong T.H., Baumgartner W.A., Johnston M.V., Marban E. (2003). Mitochondrial ATP-sensitive potassium channel activation protects cerebellar granule neurons from apoptosis induced by oxidative stress. Stroke.

[174] Kis B., Nagy K., Snipes J.A., Rajapakse N.C., Horiguchi T., Grover J., Busija D.W. (2004). The mitochondrial K(ATP) channel opener BMS-191095 induces neuronal preconditioning. Neuroreport.

[175] Nagy K., Kis B., Rajapakse N.C., Bari F., Busija D.W. (2004). Diazoxide preconditioning protects against neuronal cell death by attenuation of oxidative stress upon glutamate stimulation. J. Neurosci. Res..

[176] Sprague J.E., Huang X., Kanthasamy A., Nichols D.E. (1994). Attenuation of 3,4-methylenedioxymethamphetamine (MDMA) induced neurotoxicity with the serotonin precursors tryptophan and 5-hydroxytryptophan. Life Sci..

[177] Wang X., Baumann M.H., Xu H., Rothman R.B. (2004). 3,4-methylenedioxymethamphetamine (MDMA) administration to rats decreases brain tissue serotonin but not serotonin transporter protein and glial fibrillary acidic protein. Synapse.

[178] Wang X., Baumann M.H., Xu H., Morales M., Rothman R.B. (2005). (±)-3,4-Methylenedioxymethamphetamine administration to rats does not decrease levels of the serotonin transporter protein or alter its distribution between endosomes and the plasma membrane. J. Pharmacol. Exp. Ther..

[179] McLane M.W., McCann U., Ricaurte G. (2011). Identifying the serotonin transporter signal in western blot studies of the neurotoxic potential of MDMA and related drugs. Synapse.

[180] Cunningham J.I., Raudensky J., Tonkiss J., Yamamoto B.K. (2009). MDMA pretreatment leads to mild chronic unpredictable stress-induced impairments in spatial learning. Behav. Neurosci..

[181] O'Callaghan J.P., Miller D.B. (1993). Quantification of reactive gliosis as an approach to neurotoxicity assessment. NIDA Res. Monogr..

[182] Pubill D., Canudas A.M., Pallàs M., Camins A., Camarasa J., Escubedo E. (2003). Different glial response to methamphetamine- and methylenedioxymethamphetamine-induced neurotoxicity. Naunyn Schmiedebergs Arch. Pharmacol..

[183] Ádori C., Andó R.D., Kovács G.G., Bagdy G. (2006). Damage of serotonergic axons and immunolocalization of Hsp27, Hsp72, and Hsp90 molecular chaperones after a single dose of MDMA administration in Dark Agouti rat: Temporal, spatial, and cellular patterns. J. Comp. Neurol..

[184] Sharma H.S., Ali S.F. (2008). Acute administration of 3,4-methylenedioxymethamphetamine induces profound hyperthermia, blood-brain barrier disruption, brain edema formation, and cell injury. Ann. N. Y. Acad. Sci..

[185] Dugar A., Patanow C., O'Callaghan J.P., Lakoski J.M. (1998). Immunohistochemical localization and quantification of glial fibrillary acidic protein and synaptosomal-associated protein (mol wt 25000) in the ageing hippocampus following administration of 5,7-dihydroxytryptamine. Neuroscience.

[186] Bendotti C., Baldessari S., Pende M., Tarizzo G., Miari A., Presti M.L., Mennini T., Samanin R. (1994). Does GFAP mRNA and mitochondrial benzodiazepine receptor binding detect serotonergic neuronal degeneration in rat?. Brain Res. Bull..

[187] Rowland N.E., Kalehua A.N., Li B.H., Semple-Rowland S.L., Streit W.J. (1993). Loss of serotonin uptake sites and immunoreactivity in rat cortex after dexfenfluramine occur without parallel glial cell reaction. Brain Res..

[188] Straiko M.M.W., Coolen L.M., Zemlan F.P., Gudelsky G.A. (2007). The effect of amphetamine analogs on cleaved microtubuleassociated protein-tau formation in the rat brain. Neuroscience.

[189] Wang X., Baumann M.H., Dersch C.M., Rothman R.B. (2007). Restoration of 3,4-methylenedioxymethamphetamine-induced 5-HT depletion by the administration of l-5-hydroxytryptophan. Neuroscience.

[190] Stone D.M., Merchant K.M., Hanson G.R., Gibb J.W. (1987). Immediate and long-term effects of 3,4-methylenedioxymethamphetamine on serotonin pathways in brain of rat. Neuropharmacology.

[191] Bonkale W.L., Austin M.C. (2008). 3,4-Methylenedioxymethamphetamine induces differential regulation of tryptophan hydroxylase 2 protein and mRNA levels in the rat dorsal raphe nucleus. Neuroscience.

[192] Benmansour S., Cecchi M., Morilak D.A., Gerhardt G.A., Javors M.A., Gould G.G., Frazer A. (1999). Effects of chronic antidepressant treatments on serotonin transporter function, density, and mRNA level. J. Neurosci..

[193] Kivell B., Day D., Bosch P., Schenk S., Miller J. (2010). MDMA causes a redistribution of serotonin transporter from the cell surface to the intracellular compartment by a mechanism independent of phospho-p38-mitogen activated protein kinase activation. Neuroscience.

[194] Kirilly E. (2010). Long-term neuronal damage and recovery after a single dose of MDMA: Expression and distribution of serotonin transporter in the rat brain. Neuropsychopharmacol. Hung..

[195] O'Shea E., Orio L., Escobedo I., Sanchez V., Camarero J., Green A.R., Colado M.I. (2006). MDMA-induced neurotoxicity: Long-term effects on 5-HT biosynthesis and the influence of ambient temperature. Br. J. Pharmacol..

[196] Ádori C., Low P., Andó R.D., Gutknecht L., Pap D., Truszka F., Takács J., Kovács G.G., Lesch K.P., Bagdy G. (2011). Ultrastructural characterization of tryptophan hydroxylase 2-specific cortical serotonergic fibers and dorsal raphe neuronal cell bodies after MDMA treatment in rat. Psychopharmacology (Berl).

[197] Parrott A.C. (2001). Human psychopharmacology of Ecstasy (MDMA): A review of 15 years of empirical research. Hum. Psychopharmacol..

